# What Is the Optimal Timing of Transplantation of Neural Stem Cells in Spinal Cord Injury? A Systematic Review and Network Meta-Analysis Based on Animal Studies

**DOI:** 10.3389/fimmu.2022.855309

**Published:** 2022-03-10

**Authors:** Zhizhong Shang, Dongliang Li, Jinlei Chen, RuiRui Wang, Mingchuan Wang, Baolin Zhang, Xin Wang, Pingping Wanyan

**Affiliations:** ^1^ The First Clinical Medical School of Lanzhou University, Lanzhou, China; ^2^ Chengren Institute of Traditional Chinese Medicine, Lanzhou, China; ^3^ Department of Spine, Changzheng Hospital, Naval Medical University, Shanghai, China; ^4^ Basic Medical College, Gansu University of Chinese Medicine, Lanzhou, China; ^5^ Department of Nephrology, The Second Hospital of Lanzhou University, Lanzhou, China

**Keywords:** neural stem cells, spinal cord injury, animal study, systematic review, network meta-analysis, transplantation timing

## Abstract

**Objective:**

The optimal transplantation timing of neural stem cells in spinal cord injury is fully explored in animal studies to reduce the risk of transformation to clinical practice and to provide valuable reference for future animal studies and clinical research.

**Method:**

Seven electronic databases, namely, PubMed, Web of Science, Embase, Wanfang, Chinese Scientific Journal Database (CSJD-VIP), China Biomedical Literature Database (CBM), and China National Knowledge Infrastructure (CNKI), were searched. The studies were retrieved from inception to November 2021. Two researchers independently screened the literature, extracted data, and evaluated the methodological quality based on the inclusion criteria.

**Results and Discussion:**

Thirty-nine studies were incorporated into the final analyses. Based on the subgroup of animal models and transplantation dose, the results of network meta-analysis showed that the effect of transplantation in the subacute phase might be the best. However, the results of traditional meta-analysis were inconsistent. In the moderate-dose group of moderate spinal cord injury model and the low-dose group of severe spinal cord injury model, transplantation in the subacute phase did not significantly improve motor function. Given the lack of evidence for direct comparison between different transplantation phases, the indirectness of our network meta-analysis, and the low quality of evidence in current animal studies, our confidence in recommending cell transplantation in the subacute phase is limited. In the future, more high-quality, direct comparative studies are needed to explore this issue in depth.

## 1 Introduction

According to the Global Burden of Disease Study, the age-standardized incidence rates of spinal cord injury (SCI) is 13 cases (11–16 cases) per 100,000 people, which is equivalent to 27.04 million prevalent cases worldwide, and is on the rise (1). Also, the overall age-standardized mortality rate was 3 times higher for individuals with SCI than the general population, ranging from 27% higher for cancer to 9 times higher for infective and parasitic diseases (2). In addition, the global burden of SCI is enormous, with the highest cost among all diseases. It is estimated that all patients will cost a total of $ 2.67 billion for the first hospitalization to survive, and the subsequent costs are inestimable (3, 4). In addition to the loss of motor and sensory functions, patients with SCI will also experience a series of serious consequences, such as neuropathic pain, spasticity, and dysfunction of bladder and rectal, causing great physical and mental suffering, while SCI and SCI-related complications are difficult to recover (5). Currently, various treatments, such as anti-inflammatory medications (ketorolac, minocycline, riluzole, magnesium, etc.), decompression surgery, and good supportive management have been used clinically (6). However, these therapies merely slow down or prevent the further aggravation of injury, and it is difficult to fundamentally improve the nerve repair of patients with SCI. Therefore, therapy against SCI is still a major challenge, and recently, with the deepening research on the pathogenesis of SCI and the advances in regenerative medicine, stem cell transplantation may be an effective means to solve this problem.

Stem cells are a type of cells with self-renewal and multilineage differentiation that can originate from bone marrow, umbilical cord, adipose tissue, and neural tissue and play therapeutic roles through immunomodulation, anti-inflammation, and tissue repair (7). Many types of stem cells have shown great therapeutic potential in preclinical studies. In particular, neural stem cells (NSCs) are currently the only cells with tripotential capabilities (neurons, astrocytes, and oligodendrocytes) that can be induced and activated to differentiate into neurons to replace the missing neurons, promote vascular development, regulate inflammatory responses, and thus promote recovery from SCI (8). In addition, the source of NSCs is also rich. They can not only be obtained from various tissue components but also be differentiated from other types of stem cells such as embryonic stem cells (9), pluripotent stem cells (10), and mesenchymal stem cells (11). Therefore, NSC therapy of SCI has attracted much attention, while inconsistent results are still reported. For example, the ability of NSCs to repair SCI is limited by Nutt et al. (12). Parr et al. (13) reported that NSCs failed to significantly improve motor function after acute SCI. Karimi-Abdolrezaee et al. (14) showed that transplantation of stem cells in the subacute phase could significantly improve motor function in animals with SCI, conversely, not by transplantation in the chronic phase.

Several recent meta-analyses have shown the therapeutic potential of NSCs in SCI (15–17). However, the study has certain limitations, including the following: 1) Published studies have combined different animal models and different transplantation routes, doses, and timings. Although it has been concluded that NSCs have a significant ability to repair SCI, such a comparative “comprehensive” analysis method makes the results more heterogeneous when combined, which affects the authenticity of meta-analysis results, makes it difficult for the current results and conclusions to be transformed into clinical research, and is of limited significance for guiding clinical practice (16, 17). 2) Only the endpoint follow-up results are used for meta-analysis in the published studies, which makes the therapeutic effect of the whole process difficult to test. Moreover, it is evident that different studies have different measurement time points, and the rationality of the merger of the results at different time points is questionable (15). 3) The transplantation time of stem cells is one of the key factors to determine its targeting effect. However, few studies have explored the optimal transplantation time of NSCs (18, 19). In addition, NSCs have been initial clinical trials. However, its unsatisfactory therapeutic effect and serious complications limit further research (20). The main reason for the lack of expected results in clinical patients is that the optimal treatment strategy of NSCs remains unclear.

Therefore, we intend to comprehensively collect the published animal studies at home and abroad, explore the real effects of NSCs for SCI through traditional and network meta-analysis in node-wise manner, and further explore the optimal transplantation timing. The results will be of great value in reducing the risk of translation of animal experimental findings to the clinic, avoiding waste of experimental resources, and facilitating the development of animal studies and clinical research in the future.

## 2 Materials and Methods

### 2.1 Inclusion and Exclusion Criteria

#### 2.1.1 Subjects

The results of previous experiments based on animal models of SCI (cats, dogs, and monkeys) while conducting clinical trials were disappointing. The pathophysiology of glial scars and cysts produced during SCI in rodents (mainly rats) is more similar to humans, while at the same time being less costly and more standardized, making them the most commonly used SCI animal model to date (21). Therefore, we included rat SCI models without restricting the animal strain and modeling modality.

#### 2.1.2 Interventions

NSCs were used.

#### 2.1.3 Control

1) Positive control: comparison of different transplantation timings of NSCs; 2) Negative control: Normal saline, phosphate buffered saline (PBS), vehicle, culture medium, blank, dimethyl sulfoxide (DMSO), Dulbecco’s modified Eagle’s medium (DMEM).

#### 2.1.4 Outcome

The Basso–Beattie–Bresnahan (BBB) Locomotor Rating Scale score is a common measure of motor ability after SCI in rats (22), which ranges from 0 to 21 (spanning from complete flaccid paraplegia to normal function), and can sensitively reflect the recovery of motor function in rats. The BBB score by trained personnel is very similar enough to demonstrate the reliability and validity of the BBB score (21).

#### 2.1.5 Type of Study

Control studies were included.

#### 2.1.6 Exclusion Criteria

①Transplantation route, dose, and timing of stem cells were not reported. ②Studies that have not reported BBB scores [such as Basso Mouse Scale (BMS) scores, which are considered more suitable for assessing the recovery of motor function in mice (23)]. ③The language is non-Chinese or non-English. ④Studies that do not provide complete raw data or data cannot be extracted. ⑤Reviews, conferences, commentary articles.

#### 2.1.7 Data Selection

Early stage of inflammation is mainly composed of neutrophils (peak at 1 day after injury), macrophages/microglia (peak at 7 days after injury), and T cells (peak at 9 days after injury) (24). In addition, studies on rats showed that transplanting macrophages with an M2 phenotype promoted nerve regeneration and improved functional recovery after SCI in rats; also, macrophages peaked again 60 days after injury (24–26). Therefore, we selected the research data of 1 week and 8 weeks after stem cell therapy (the period with the strongest inflammatory response) for analysis of results. Moreover, glial scar composed of astrocytes can form a physical barrier to inhibit axon growth. Three weeks after injury is a critical period of astrocyte scar maturation (21). Therefore, we selected the research data of 3 weeks after stem cell therapy for analysis of results. Previous research has shown that recovery of motor function after SCI in rats appeared to reach a plateau around 5 weeks (27, 28). Therefore, we selected the research data of 5 weeks after stem cell therapy for analysis of results.

### 2.2 Retrieval Strategy

We searched scientific databases such as PubMed, Ovid-Embase, Web of Science, China National Knowledge Infrastructure (CNKI), Chinese Scientific Journal Database (CSJD-VIP), Wanfang Database, and China Biomedical Literature Database (CBM). The relevant literature was retrieved from inception to November 2021. The search terms were (Spinal cord injury OR Spinal injury OR Spinal Cord Trauma OR Spinal Cord Transection OR Spinal Cord Laceration OR Post-Traumatic Myelopathy OR Spinal Cord Contusion) AND (Neural stem cell OR Nerve stem cell OR Neuronal stem cells). **Supplementary Table S1** describes the detailed search strategies of each database.

### 2.3 Literature Screening and Data Extraction

Two trained researchers selected the papers and stringently extracted the data based on the inclusion/exclusion criteria, and the selections were cross-checked. In the case of disagreement, a third researcher settled the conflict with a common consensus. Data were extracted according to the pre-established full-text data extraction checklist, including 1) basic characteristics of studies such as authors, publication years, type of study, baseline characteristics of rats (gender, age, weight), sample size, modeling method, source of NSCs, transplantation route, dose, timings, controls; 2) key elements of bias risk assessment; 3) outcome measures: BBB score.

### 2.4 The Risk of Bias Among Included Studies

Based on SYRCLE’s Risk of Bias tool for animal studies (29), 2 trained researchers independently evaluated and cross-checked the inherent risk of bias in the included studies, covering selection bias, implementation bias, measurement bias, follow-up bias, report bias, and other biases from a list of 10 questions or tools. A difference in opinions was negotiated or decided by a third party. The answer to the assessment questions (tools) should be either “yes” that indicated a low risk of bias or “no” that indicated a high risk of bias. For unclear items, an answer with “unclear” was assigned.

### 2.5 Statistical Analysis

#### 2.5.1 Traditional Meta-Analysis

We used STATA 16 software in order to perform a traditional meta-analysis of direct comparison of BBB score results for NSCs with negative controls. Weighted mean difference (WMD) was regarded as the effect analysis statistic and provided its 95% CI. The heterogeneity among included studies was analyzed by χ^2^ test (test level was α = 0.1) and was quantitatively judged by I^2^. If there was no statistical heterogeneity, meta-analysis was performed using fixed-effects model; otherwise, sources of heterogeneity were further analyzed, and after the influence of obvious clinical heterogeneity is excluded, the random-effects model was used for meta-analysis. The α level was set at 0.05.

#### 2.5.2 Network Meta-Analysis

GeMTC-0.14.3 software based on the Bayesian model was used for statistical analysis. The software used Markov chain Monte Carlo (MCMC) to prioritize and evaluate the data to achieve network meta-analysis. The deviation information criterion (DIC) value of the random-effects model and fixed-effects model were compared to analyze the fitting degree of the model. The network meta-analysis used the concordance model, which was statistically significant. The node analysis model was used for the inconsistency test; if P > 0.05, there was no evidence to prove the direct and indirect comparison inconsistencies. The convergence of network meta-analysis was tested by the potential scale reduction parameter (PSRF). If PSRF was close to 1, the convergence of this study was good, and the conclusion of the meta-analysis was reliable. Also, the network group commands were used for data preprocessing based on STATA 16 software to compare the outcome indicators of the network relationship between the intervention measures.

#### 2.5.3 Subgroup Analysis

**Figure 1:**
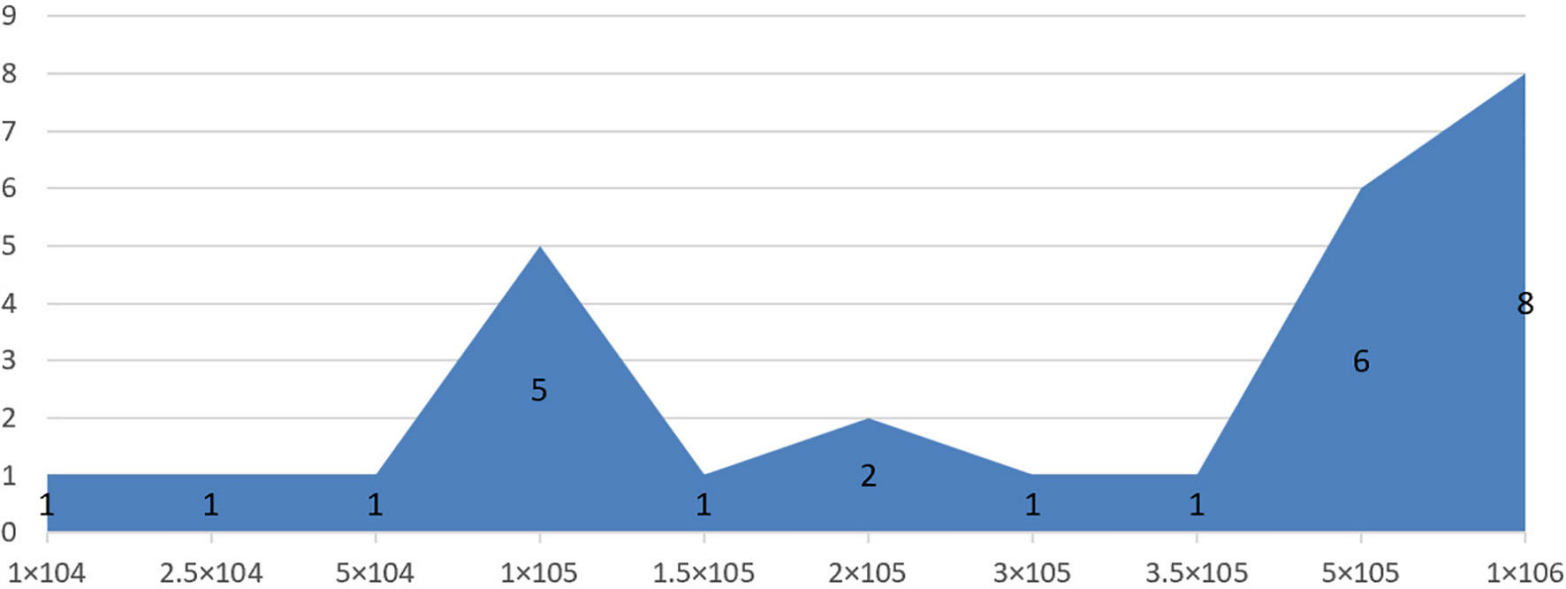
Area chart of transplantation dose of neural stem cells (NSCs).

**Figure 2:**
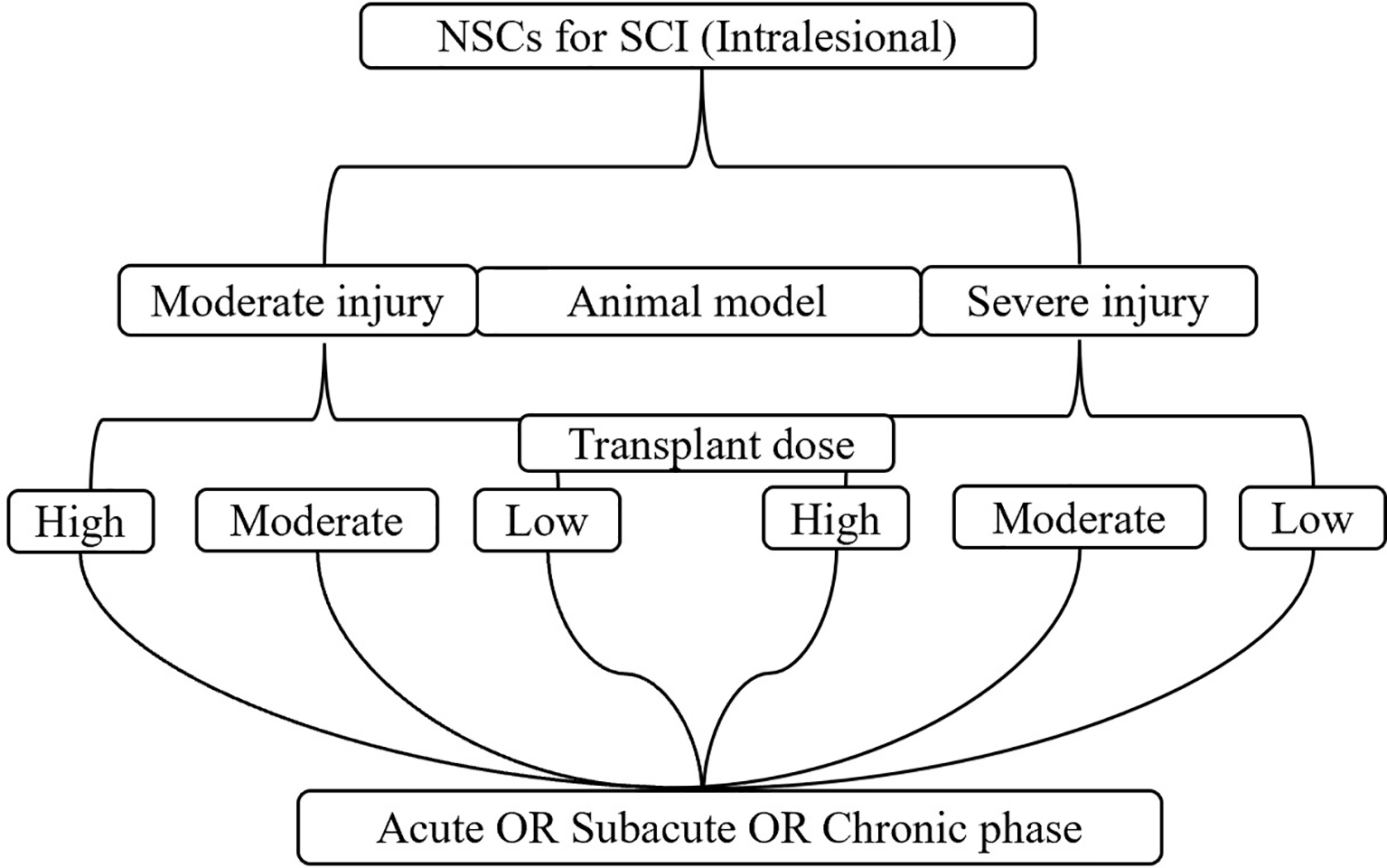
The process of subgroup analysis.

To avoid the impact of mixed factors (e.g., different model types, transplantation routes, doses, and timings) on the results of the meta-analysis, while reducing the heterogeneity between the included studies, subgroup analysis was performed. 1) Animal model: moderate injury (compression and contusion) (30) and severe injury (transection). 2) Transplantation dose: high dose (≥1 × 10^6^), moderate dose (1 × 10^5^–1 × 10^6^), and low dose (≤1 × 10^5^). The basis of dose division is shown in **Figure 1**. 3) Transplantation route: intralesional transplantation. Since there were only 2 studies on the subarachnoid space and tail vein transplantation, the results of the meta-analysis were easily affected by single studies and small sample sizes, so they were excluded. 4) Transplantation timing: Acute phase (≤3 days), subacute phase (3–7 days, including 7 days, excluding 3 days), chronic phase (>7 days). The process of subgroup analysis is shown in **Figure 2**.

## 3 Results

### 3.1 Literature Search Results

**Figure 3:**
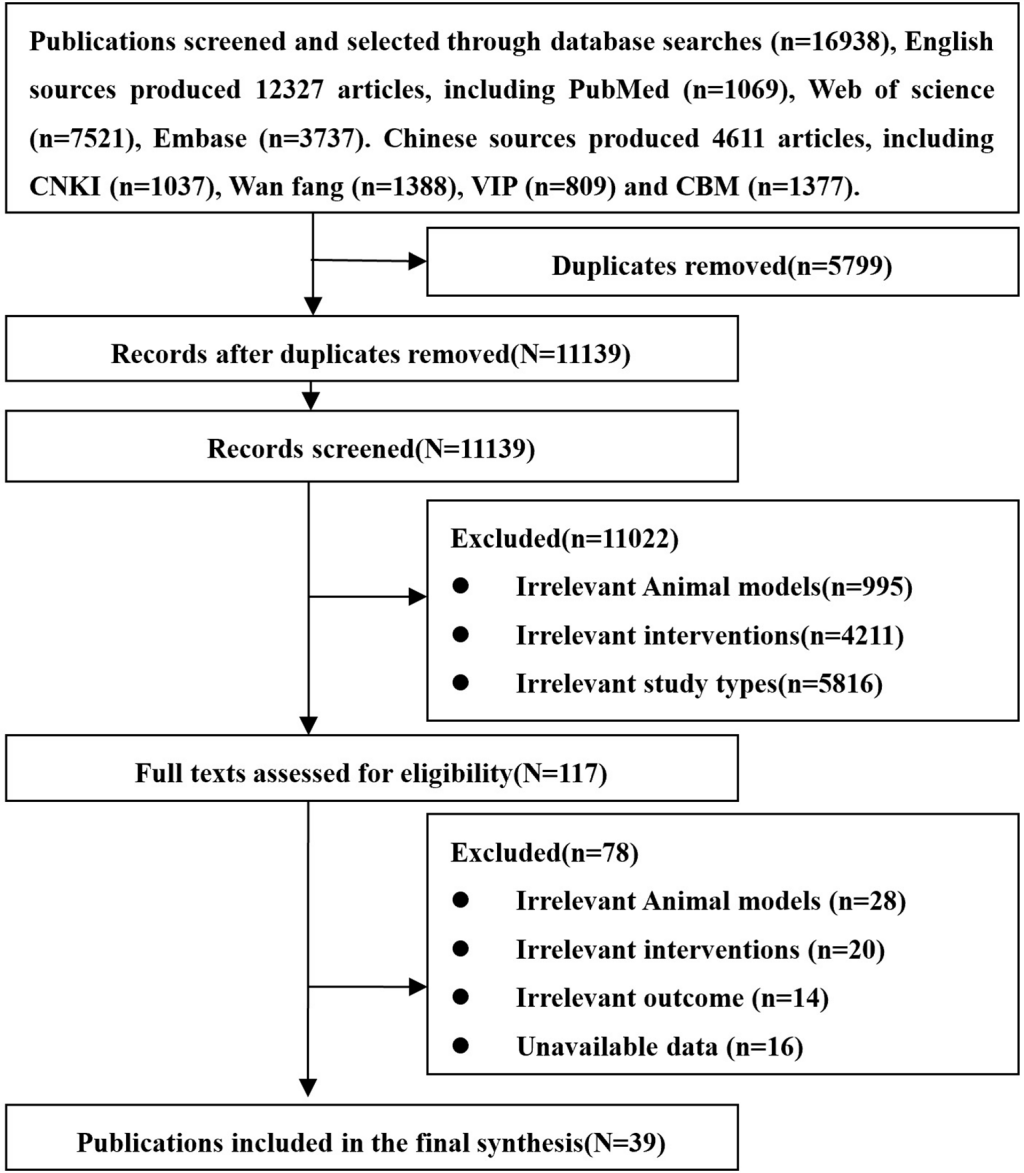
Flowchart of literature screening.

A total of 16,938 related articles including 12,327 English and 4,611 Chinese records were obtained. After excluding the literature based on the exclusion criteria, eventually 39 studies were included. The studied 39 studies include 26 English and 13 Chinese articles. The entire screening process is summarized in **Figure 3**.

### 3.2 Basic Information for Inclusion in the Study

Of the 39 included studies, only one was a controlled experience, and the rest were randomized controlled experiences. The varieties of rats included Sprague-Dawley (SD) rats (27 studies), Wistar rats (10 studies), and Long-Evans hooded rats (2 studies). The gender of rats included male (11 studies), female (17 studies), and half male and half female (3 studies), and 8 studies did not report gender of rats. The weight of rats was between 180 and 350 g, the age was between 4 and 16 weeks, and the sample size was between 10 and 80. The modeling methods included compression (4 studies), contusion (23 studies), and transection (12 studies). The sources of NSCs included brain tissue of SD rats and Wistar rats, human or cynomolgus aborted embryos, etc. The transplantation routes of NSCs were all intralesional transplantation, with the transplantation doses between 1 × 10^4^ and 1 × 10^6^, and the transplantation timings between 0 and 10 days after modeling. Negative controls included blank, DMEM, DMSO, PBS, saline, and Hanks Balanced Salt Solution. The basic information of the included studies is detailed in **Supplementary Table S2**.

### 3.3 Risk of Bias Assessment Results

**Figure 4:**
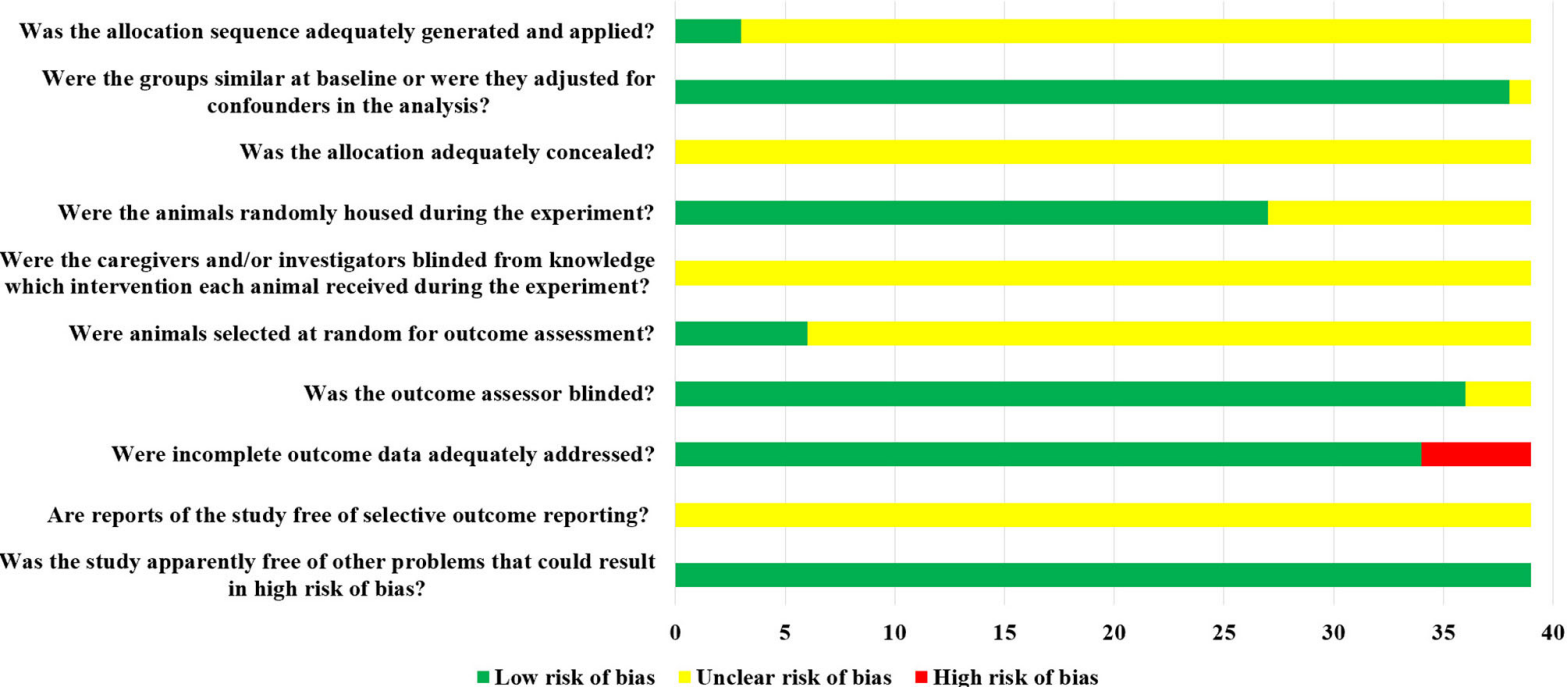
Risk of bias assessment results.

Of the 39 studies included, 38 were randomized controlled experiences, of which only 3 studies described specific randomized grouping methods but did not specify whether covert groupings were implemented. One study reported only the species of animals, which made it impossible to judge whether its baseline characteristics were balanced. The remaining 38 studies clearly reported the baseline characteristics of animals. Among them, 27 studies randomized the placement of animals during the experiment. Due to limited information provided by the included studies, we were unable to determine whether or not blinding of animal breeders and/or researchers was performed. Only 6 studies showed that animals were randomly selected to measure the results. Among them, 36 studies blinded the evaluators of the results. There were 5 studies in which animals died during the experiment. Although no research protocols were available for any of the studies, all expected results were clearly reported. The risk of bias assessment for all studies is detailed in **Figure 4**.

### 3.4 Meta-Analysis Results

#### 3.4.1 Traditional Meta-Analysis Results of Moderate Injury

1) High-dose group: In the first week and the third week, there was no significant difference in BBB scores between the NSC group and the negative control group in the acute phase [Week 1: WMD = 0.63 (-0.06, 1.31); Week 3: WMD = 2.53 (-0.56, 5.62)]. Similarly, in the first and fifth weeks, there was no significant difference in BBB scores between the NSC group and the negative control group in the chronic period [Week 1: WMD = 0.38 (-0.41, 1.17); Week 5: WMD = -0.24 (-0.78, 0.30)].
2) Moderate-dose group: In the first week, there was no significant difference in BBB scores between the NSC group and the negative control group in the acute phase [Week 1: WMD = 1.86 (-0.62, 4.34)]. In the fifth week, there was no significant difference in BBB scores between the NSC group and the negative control group in the chronic phase [Week 5: WMD = 1.77 (-1.20, 4.73)]. In the first, third, and eighth week, there was no significant difference in BBB scores between the NSC group and the negative control group in the subacute phase [Week 1: WMD = 4.10 (-3.62, 11.82); Week 3: WMD = 4.81 (-2.27, 11.89); Week 8: WMD = 6.92 (-0.69, 14.52)].
3) Low-dose group: In the first and third week, there was no significant difference in BBB scores between the NSC group and the negative control group in the chronic phase [Week 1: WMD = 0.66 (-0.83, 2.14); Week 3: WMD = 1.53 (-0.38, 3.43)].

#### 3.4.2 Traditional Meta-Analysis Results of Severe Injury

1) High-dose group: In the first week, there was no significant difference in BBB scores between the NSC group and the negative control group in the acute phase [Week 1: WMD = 0.54 (-0.02, 1.10)].
2) Moderate-dose group: The BBB scores in the NSC group were significantly higher than those in the negative control group.
3) Low-dose group: In the first week, there was no significant difference in BBB scores between the NSC group and the negative control group in the subacute phase [Week 1: WMD = 0.25 (-0.34, 0.83)].

In addition to the above results, the BBB scores in the NSC group were significantly higher than those in the negative control group both in moderate and severe injury models. See **Supplementary Table S3**.

#### 3.4.3 Network Meta-Analysis Results of Moderate Injury

**Figure 5:**
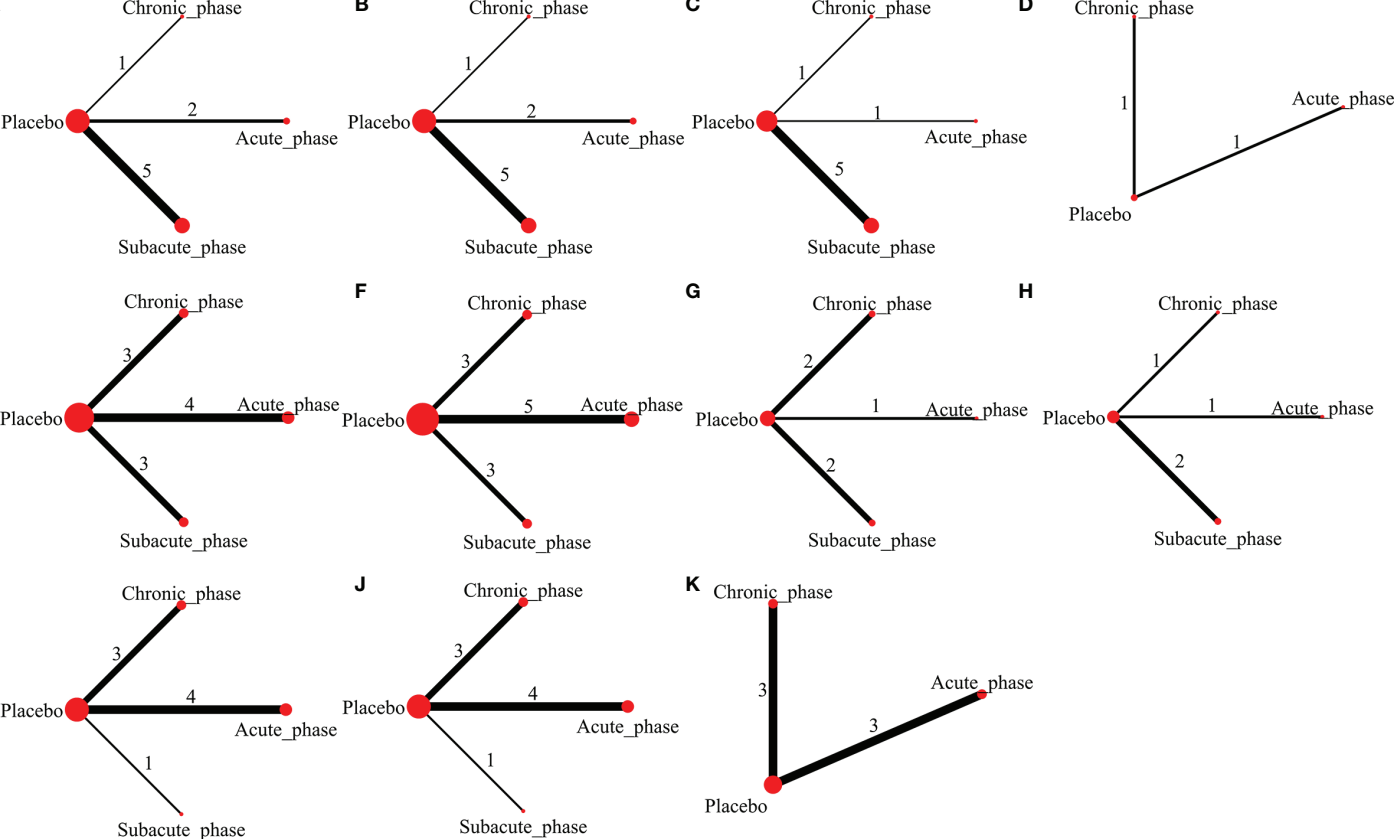
Evidence network plots of moderate injury. (**A–D**, results of 1, 3, 5, and 8 weeks of high-dose group; **E–H**, results of 1, 3, 5, and 8 weeks of moderate-dose group; **I–K**, results of 1, 3, and 5 weeks of low-dose group).

**Figure 6:**
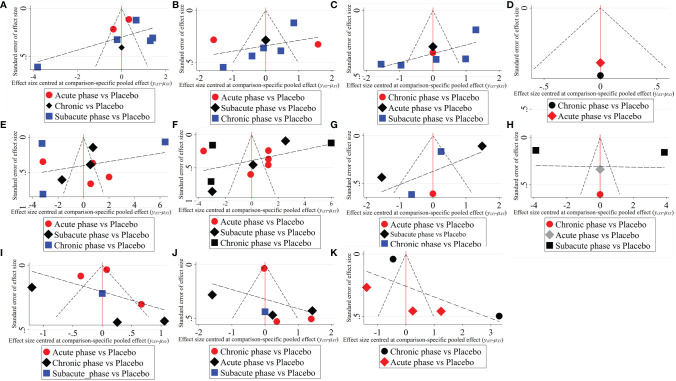
The comparison-corrected funnel plots of moderate injury. (**A–D**, results of 1, 3, 5, and 8 weeks of high-dose group; **E–H**, results of 1, 3, 5, and 8 weeks of moderate-dose group; **I–K**: results of 1, 3, and 5 weeks of low-dose group).

**Figure 7:**
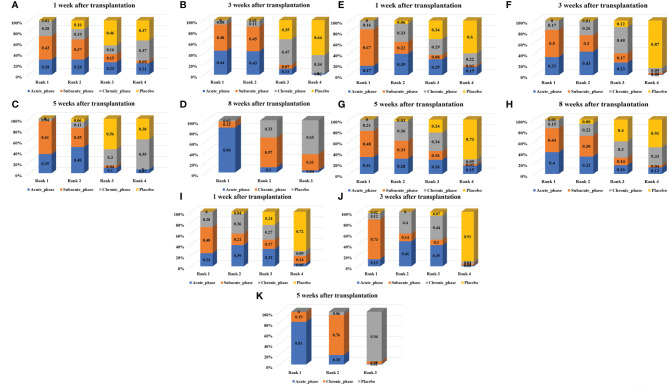
Ranking results of moderate injury. (**A–D**, results of 1, 3, 5, and 8 weeks of high-dose group; **E–H**, results of 1, 3, 5, and 8 weeks of moderate-dose group; **I–K**: results of 1, 3, and 5 weeks of low-dose group).

1) High-dose group: A total of 8 studies were included for network meta-analysis. Evidence network plots at different time points showed that there is no direct comparison between different transplantation phases, along with the largest number of studies in the subacute phase (**Figures 5A**–**D**). The comparison-corrected funnel plots at different time points were asymmetric, indicating that publication bias and small sample effect may exist (**Figures 6A**–**D**). The results of network meta-analysis at different time points showed that there was no statistically significant difference in the improvement of the motor function at different transplantation phases of NSCs (**Table 1**). The ranking results at different time points all showed that the ability to promote the recovery of motor function was in the order of subacute phase > acute phase > chronic phase (**Figures 7A**–**D**).
2) Moderate-dose group: A total of 11 studies were included for network meta-analysis. Evidence network plots at different time points showed that there is no direct comparison between different transplantation phases, along with the largest number of studies in acute phase (**Figures 5E**–**H**). The comparison-corrected funnel plots at different time points were asymmetric, indicating that publication bias and small sample effect may exist (**Figures 6E**–**H**). The results of network meta-analysis at different time points showed that there was no statistically significant difference in the improvement of the motor function at different transplantation phases of NSCs (**Table 1**). The ranking results at different time points all showed that the ability to promote the recovery of motor function was in the order of subacute phase > acute phase > chronic phase (**Figures 7E**–**H**).
3) Low-dose group: A total of 8 studies were included for network meta-analysis. Evidence network plots at different time points showed that there is no direct comparison between different transplantation phases, along with the largest number of studies in the chronic phase (**Figures 5I**–**K**). The comparison-corrected funnel plots at different time points were asymmetric, indicating that publication bias and small sample effect may exist (**Figures 6I**–**K**). The results of network meta-analysis at different time points showed that there was no statistically significant difference in the improvement of the motor function at different transplantation phases of NSCs (**Table 1**). The ranking results showed that the ability to improve the motor function was in order from high to low: first week: subacute phase > chronic phase > acute phase; third week: subacute phase > acute phase > chronic phase; fifth week: acute phase > chronic phase; eighth week: research data without subacute and chronic phases (**Figures 7I**–**K**).

**Table 1:** Network meta-analysis results of moderate injury.

High dose—first week			Moderate dose—first week		
Acute phase			Acute phase		
0.30 (-4.42, 4.94)	Chronic phase		0.32 (-6.35, 7.27)	Chronic phase	
-0.49 (-3.72, 2.74)	-0.76 (-5.01, 3.46)	Subacute phase	-2.30 (-9.03, 4.69)	-2.62 (-9.90, 4.77)	Subacute phase
**High dose—third week**			**Moderate dose—third week**		
Acute phase			Acute phase		
1.93 (-2.44, 6.34)	Chronic phase		1.29 (-4.93, 7.09)	Chronic phase	
-0.09 (-3.12, 2.83)	-2.04 (-6.05, 1.95)	Subacute phase	-0.63 (-6.60, 5.47)	-1.87 (-8.63, 5.04)	Subacute phase
**High dose—fifth week**			**Moderate dose—fifth week**		
Acute phase			Acute phase		
2.30 (-3.00, 7.53)	Chronic phase		0.15 (-5.26, 5.55)	Chronic phase	
-0.58 (-4.74, 3.60)	-2.93 (-7.04, 1.27)	Subacute phase	-0.71 (-6.03, 4.88)	-0.83 (-5.11, 3.48)	Subacute phase
**High dose—eighth week**			**Moderate dose—eighth week**		
Acute			Acute phase		
2.53 (-2.85, 7.94)	Chronic phase		4.29 (-16.55, 24.24)	Chronic phase	
			-0.67 (-18.63, 16.58)	-4.88 (-22.69, 13.10)	Subacute phase
**Low dose—first week**			**Low dose—third week**		
Acute phase			Acute phase		
-0.03 (-1.54, 1.66)	Chronic phase		0.09 (-2.32, 2.46)	Chronic phase	
-0.23 (-2.43, 1.98)	-0.21 (-2.69, 2.07)	Subacute phase	-1.27 (-4.82, 2.19)	-1.32 (-4.94, 2.17)	Subacute phase
**Low dose—fifth week**					
Acute phase					
1.62 (-2.77, 6.09)	Chronic phase				

#### 3.4.4 Network Meta-Analysis Results of Severe Injury

**Figure 8:**
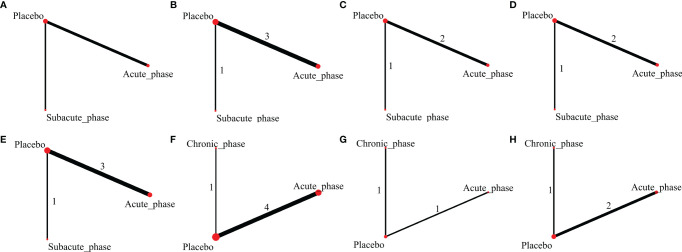
Evidence network plots of severe injury. (**A–D**, results of 1, 3, 5, and 8 weeks of high-dose group; **E–H**, results of 1, 3, 5, and 8 weeks of moderate-dose group).

**Figure 9:**
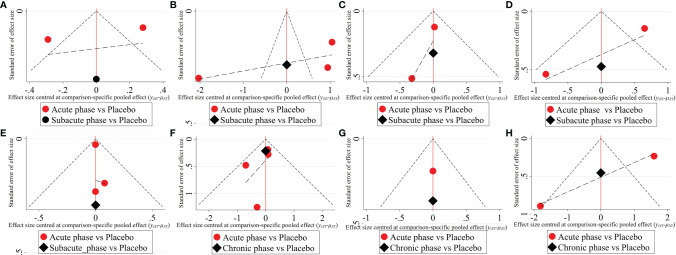
The comparison-corrected funnel plots of severe injury. (**A–D**, results of 1, 3, 5, and 8 weeks of high-dose group; **E–H**, results of 1, 3, 5, and 8 weeks of moderate-dose group).

**Figure 10:**
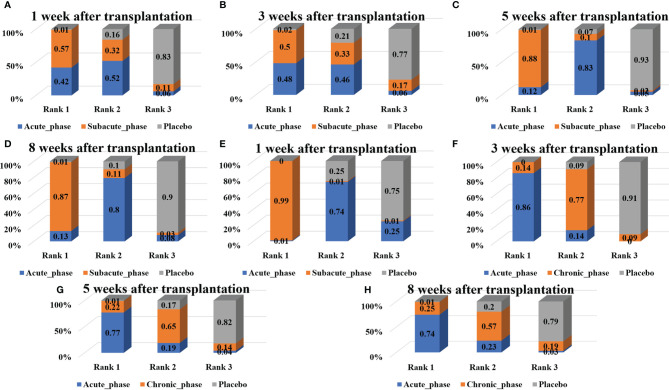
Ranking results of severe injury. (**A–D**, results of 1, 3, 5, and 8 weeks of high-dose group; **E–H**, results of 1, 3, 5, and 8 weeks of moderate-dose group).

1) High-dose group: A total of 4 studies were included for network meta-analysis, all of which were acute or subacute phases. Evidence network plots at different time points showed that there is no direct comparison between different transplantation phases, along with the largest number of studies in the acute phase (**Figures 8A**–**D**). The comparison-corrected funnel plots at different time points were asymmetric, indicating that publication bias and small sample effect may exist (**Figures 9A**–**D**). The results of network meta-analysis at different time points showed that there was no statistically significant difference in the improvement of the motor function at different transplantation phases of NSCs [acute phase vs. subacute phase, Week 1: WMD = -0.11 (-1.52, 1.30); Week 3: WMD = -0.01 (-6.10, 5.75); Week 5: WMD = -1.52 (-4.97, 2.15); Week 8: WMD = -1.52 (-4.97, 2.15)]. The ranking results at different time points all showed that the ability to promote the recovery of motor function was in the order of subacute phase > acute phase (**Figures 10A**–**D**).
2) Moderate-dose group: A total of 5 studies were included for network meta-analysis, all of which were acute or subacute phases. Evidence network plots at different time points showed that there is no direct comparison between different transplantation phases, along with the largest number of studies in the acute phase (**Figures 8E**–**H**). The comparison-corrected funnel plots at different time points were asymmetric, indicating that publication bias and small sample effect may exist (**Figures 9E**–**H**). The results of network meta-analysis in the first week showed that the effect of transplantation in the subacute phase is significantly better than that in the acute phase [acute phase vs. subacute phase: WMD = -1.26 (-2.20, -0.33)]. In the third, fifth, and eighth week, only the acute and the chronic phases were studied. The results showed that there was no statistically significant difference in the improvement of the motor function at different transplantation phases of NSCs [acute phase vs. chronic phase, Week 3: WMD = 0.60 (-1.00, 2.50); Week 5: WMD = 1.42 (-3.90, 6.71); Week 8: WMD = 2.78 (-8.78, 15.10)]. The ranking results at different time points all showed that the ability to promote the recovery of motor function was in the order of subacute phase > acute phase > chronic phase (**Figures 10E**–**H**).

## 4 Discussion

### 4.1 Overview of Evidence

The powerful therapeutic efficacy of stem cells must be demonstrated in animal models before starting a translational clinical trial, and issues such as the timings of transplantation and the risk of side effects should be addressed to reduce the risk of their transformation to clinical practice. The systematic review of animal studies is a powerful means to solve this problem. It can synthesize the results of multiple studies, fully explore the real effect of a certain intervention, and simultaneously evaluate the feasibility and risk of its clinical transformation.

In the traditional meta-analysis, through subgroup analysis of animal models and transplantation routes and doses, we found that the effects of high-dose cell transplantation in the subacute phase are significantly better than those of the negative control group both in the moderate injury model or the severe injury model. On the contrary, there was no significant difference between the effect of high-dose cell transplantation and the negative controls in the acute and chronic phases, which fully suggests that subacute transplantation may be the optimal period of cell transplantation. However, in the moderate-dose and low-dose cell transplantation, there was no significant statistical difference between the transplantation results of each phase and the negative control group. The possible reason is that the transplantation dose of cells did not reach the therapeutic dose. Therefore, no matter which phase of transplantation was carried out, the improvement of motor function in animals with SCI was not significant. After SCI, endogenous neurotrophic factors, such as brain-derived neurotrophic factor (BDNF) and glial-derived neurotrophic factor (GDNF), begin to express and decrease to normal levels after lasting 3–4 days (31, 32). Therefore, the microenvironment inside the injured tissues in the subacute phase is more suitable for the survival and differentiation of exogenous cells than those in the acute and chronic phases. For example, Keirstead et al. (33) induced differentiation of human embryonic stem cells into oligodendrocytes [oligodendrocyte progenitor cells (OPCs)] and transplanted them to the SCI site and found that the rats that received cell transplantation on the seventh day after injury showed significant remyelination and functional recovery, while rats that received OPCs at 10 months did not show significant functional recovery. Its pathological findings revealed extensive astrogliosis in rats undergoing cell transplantation in the chronic phase and that axons were gradually phagocytosed by astrocytes; thus, the axons could not be connected after exogenous cells were implanted (33). Given the few studies comparing different transplantation timings of NSCs, we indirectly compare the therapeutic effects of different transplantation timings through a network meta-analysis.

In the network meta-analysis, although there was a lack of relevant phase data in some subgroups, the results obtained from the subgroup analysis of different transplantation doses are highly consistent, which showed that the transplantation effect in the subacute phase was better than that in the acute phase, and the transplantation effect in the acute phase was better than that in the chronic phase. The results of the network meta-analysis once again proved the advantages of subacute transplantation. Results from *in vivo* animal experiments showed that the infiltration of neutrophils [polymorphonuclear neutrophils (PMNs)] and macrophages/microglia after SCI exacerbates the inflammation (24, 34–36). Similarly, central nervous system-reactive T cells can also cause loss of function of the injured spinal cord by damaging axons and demyelination (24, 37). On the contrary, macrophages/microglia and T cells can also secrete nutritional factors to promote neuroprotection and nerve regeneration after SCI over time (24, 38), so as to realize the transformation from pro-inflammatory to anti-inflammatory (24, 39). Among them, neutrophils, as the first immune cells to infiltrate the injured spinal cord, reach a peak on the first day after injury, then gradually decline and persist in the body for up to 6 months. Therefore, the transplanted cells in the acute phase may be attacked by neutrophils and lose their repair function (24). Macrophages/microglia can be detected on the third day after injury and reach a peak on the seventh day (24); that is, the infiltration of macrophages/microglia is relatively low in the subacute phase (3–7 days) and gradually increases. Therefore, the cells transplanted in the subacute phase are less attacked by immune cells *in vivo*. At the same time, the study by Li et al. (40) also showed that the *in vivo* is very unfavorable for transplanted cells in the acute phase due to the activation of inflammatory cascade, cell death, and upregulation of inflammatory mediators. On the contrary, the level of macrophage infiltration in the subacute phase is relatively low, and the *in vivo* at this time is more conducive to the survival of transplanted cells (40). For chronic transplantation, although the infiltration of neutrophils and macrophages/microglia has decreased significantly, the infiltration of T cells begin to increase and gradually reach the peak on the seventh day after injury, while the infiltration of T cells is not detected within the first 7 days after SCI (24). Therefore, transplantation in the acute and subacute phases can be protected from the attack of T cells. Moreover, a period of time after SCI, the damage signal generated at the injury site is gradually lost, and at the same time, the vascular bed at the injury site is seriously damaged. Therefore, the injured tissues lack the signal to attract exogenous stem cells and the vascular bed needed for cell planting, resulting in low cell survival rate of chronic transplantation. This conclusion is supported by the findings of Parr et al. (13), who showed that cell survival was significantly higher in the subacute phase than that in the acute and chronic phases. In contrast, the therapeutic effect of NSCs was not statistically different by the timing of transplantation, as shown by Kumamaru et al. (41). Meanwhile, transplanted cells in the chronic phase were more capable to differentiate into neurons/oligodendrocytes and to produce nutritional factors. They believe that the main reason for the failure of transplantation in the chronic phase is the refractory state of chronic SCI, such as the formation of glial scars (41). In short, the role of macrophages/microglia in SCI has always been controversial, and it is not clear when the functional change from pro-inflammatory to anti-inflammatory occurs (24, 42). Therefore, further study of the changes of internal environment and various cytokines in animals after SCI is essential to better explore and understand the optimal period of cell transplantation.

Looking back at the previously published research, the traditional meta-analysis results of Yousefifard et al. (15) showed that the effect size of transplantation in the acute phase [standardized mean difference (SMD) = 1.8 (1.36, 2.24)] is slightly higher than that in the subacute phase [SMD = 1.38 (1.08, 1.67)]. This is contrary to our conclusion. The possible reason is that the period division of cell transplantation after SCI by Yousefifard et al. (15) is different from that of our research. They defined cell transplants performed 3–10 days post SCI as subacute phase and, therefore, their subacute phase actually included the results of the subacute and chronic phases of our study. In our study, the effect of chronic phase transplantation was the worst. Moreover, the major limitation of their research is that when subgroup analysis of transplantation period is carried out, great differences in animal models and transplantation routes and doses in different studies are not considered. Therefore, the analysis results may be biased by the transplantation routes and doses. On the contrary, we only included the studies of intralesional transplantation, while dividing the severity of the models and the doses of transplantation, based on which we further compared the transplantation effect of different transplantation phases. At the same time, they combined the data of different scores (BBB score, BMS score) during the subgroup analysis of the transplantation phase and used SMD as the effect size. The combined analysis of different scoring standards will lead to great heterogeneity among studies, and SMD is also prone to exaggerate the therapeutic effect of interventions. However, we only included the BBB score and used WMD for the combined analysis of the results, and the results obtained were more reliable.

It is worth noting that the results of our traditional meta-analysis are inconsistent with those of the network meta-analysis, which may be affected by cell transplantation doses (too low transplantation doses do not significantly improve motor function in animals with SCI at any transplantation phase). In view of the lack of direct comparison between different transplantation phases, and the results of the network meta-analysis are also obtained through indirect comparison, it is necessary to be very cautious about the conclusion that stem cell transplantation in the subacute phase is better. Considering the feasibility of clinical transformation, SCI usually occurs outside the hospital. It takes a certain time to send patients to the hospital for first aid, conduct a series of examinations to assess the condition, and prepare cell products. Therefore, it is impossible for clinical patients to transplant stem cells immediately after SCI like experimental animals. In summary, we believe that cell transplantation in the subacute phase is better and more in line with clinical practice.

### 4.2 Quality of Evidence

#### 4.2.1 Heterogeneity

1) The heterogeneity of the included studies was reduced as much as possible by dividing the animal model and cell transplantation routes, doses, and timings into subgroups. Therefore, our results are relatively reliable.
2) Our network meta-analysis selected a well-known BBB score for evaluating motor function after SCI. Although trained researchers have been highly consistent on BBB scores (21), given the differences in training and experimental background and conditions, the scores of the same degree of injury by different researchers must show differences, which may greatly affect the authenticity of the meta-analysis results, thereby further affecting the reliability of the research conclusions.

#### 4.2.2 Internal Authenticity

1) Randomization: The lack of randomization and allocation concealment in animal studies will affect the reliability of the results by changing the effect size (43–45). Although 97.44% (38/39) of the studies were randomized controlled experiments, only 7.69% (3/39) of the studies reported specific randomization methods, and no study has reported whether allocation concealment was implemented. Therefore, there was a certain selection bias in the included studies.
2) Blinding: The blinding method in animal experiments includes blinding the experimental animals, experiment implementers, and result measurers. Studies have shown that the lack of blinding can lead to an exaggeration of the effect size (43, 44). Although blinding is not required in animal research, and researchers in most studies are also animal breeders, blinding may be required during the intervention and measurement phases of animal research to reduce performance and detection bias and increase the authenticity of the results (46). Of the 39 included studies, although 36 studies blinded the result evaluators, the information provided by the included studies was limited, and it was impossible to judge whether they blinded animal breeders and researchers. Therefore, future research should pay more attention to the application of blinding in the experimental design, and at the same time, more experimental details should be provided to improve the report quality of animal experiments.
3) Results report: All included studies clearly reported all expected results in their research methods and results, but they were unable to obtain the protocols of the study, and it was impossible to finally judge whether to follow the protocols and report all the results without bias. The selective reporting of animal experimental results may lead to publication bias, thus affecting the reliability of the systematic review, and even come to opposite conclusions (47). Although it is very difficult to register an animal experiment protocol, we still encourage animal experiment researchers to prospectively register experiment protocols and, at the same time, use the original data as an online appendix to improve the transparency and quality of animal studies (48).
4) Publication bias: Studies with positive results are usually more likely to be published than those with negative or ineffective results (49). Publication bias may be more serious in animal studies (50). Therefore, if a systematic review does not include unpublished research, it is likely to overestimate the effect of intervention. We can conclude by making comparison-corrected funnel plots that there may be a certain degree of publication bias in this field. However, due to the small number of studies on publication bias detection (usually more than 10 studies; the test results of publication bias are more reliable), the current results cannot fully determine the existence of publication bias. In conclusion, in the field of experimental research, it is necessary to take measures to promote data sharing and develop policies to encourage and require journals to publish studies with negative or neutral results in order to avoid the “file drawer” effect and reduce the impact of publication bias (51).

#### 4.2.3 External Authenticity

External authenticity refers to the extent to which clinical trial results can be reproduced repeatedly in the target population and daily population (52). In animal experiments, the external authenticity mainly includes the repeatability of the animal experiments and the feasibility of transforming the research results to the clinic.

1) Animal models of SCI are usually caused by contusion with heavy objects on the surface of the spinal cord or precise transverse injuries caused by scissors after laminectomy. However, SCI in clinical patients is usually caused by motor vehicle accidents (38%), falls (>22%), violence (13.5%), and sports and recreational accidents (9%) (21). Therefore, the conditions of patients with SCI are more complicated.
2) Clinically, 60% of injury of patients occurs in the cervical spine, followed by the thoracic spine (32%) and lumbosacral spine (9%) (53). However, a rat model of SCI established at the cervical level can lead to simultaneous paralysis of the forelimb and hindlimb. At the same time, due to the difficulty of operation, animal models of SCI in the thoracic or lumbar spine are often used in animal experiments that are more technically feasible (21).
3) Whether the staging standards in animal experiments can be replicated or analogized to clinical research is a huge challenge. For example, in animal models, SCI reaches a plateau at about 5 weeks; however, the natural recovery of humans is considered to reach a plateau at 6–12 months after injury (21).
4) In fact, Crl : NIH-Foxn1 rnu nude rats are the most rigorous research objects used to evaluate the true efficacy of NSCs. However, the rats currently used in animal experiments are SD, Lewis, or Wistar rats, and nude rats have not been studied.
5) Compared to clinical studies, there are significant differences in culture and storage conditions, characterization pipelines, and transplantation conditions of the laboratory. In addition, common methods of evaluating grafts, such as immunohistochemistry and biofluorescent labeling, are usually not possible in humans (54).
6) In the course of clinical SCI, the efficacy of stem cells can be affected by the patient’s medical history and internal or external physical conditions. For example, aging and diabetes may lead to impaired stem cell proliferation, decreased angiogenesis, and reduced wound healing, while animal experiments are difficult to simultaneously simulate various human physical conditions (55).
7) Longer follow-up results can predict the motor function recovery trajectory of SCI animals more comprehensively, which can reduce the number of subjects required for subsequent clinical trials and better guide clinical practice. However, few preclinical studies extend the follow-up time to 2 months after cell transplantation. Therefore, future animal experiments should extend the follow-up time and further observe the long-term therapeutic effects of stem cells.

#### 4.2.4 Species Differences Between Humans and Rats

Translating experimental results in rats into clinical studies is very difficult due to differences in neurological function and anatomy (56). The differences in SCI between humans and rats are mainly manifested in the following aspects. 1) In rats, reactive astrocytes aggregate at the edge of the injury 1–3 weeks after injury to begin forming a glial scar and gradually mature. Observations of human SCI found that this scarring occurs later (46 months after injury) (57, 58). 2) Indeed, experimental results from rodent studies that report improved axonal growth (e.g., because of axons bridging the lesion site) might misinform us because the volumes of gray matter that need reinnervation are much larger in humans than that in rats. 3) Spontaneous recovery in humans is not considered to reach a plateau until 6–12 months after injury. On the contrary, rats returned to the typical plateau at 6–8 weeks after injury (21). 4) The redundant prominent motor tracts in rats allow for effective rerouting around the injury. The enlarged corticospinal tract in primates, along with the reduced rubrospinal tract in humans may impact the efficacy of plasticity in human (59).

Although there is an unbridgeable species gap between humans and rats, there are many issues, such as the timing, dose, route, and side effects of stem cell transplantation, which can be effectively explored in rats, thereby improving successful extrapolation to human probability on the body. Therefore, rat models of SCI are essential to guide future clinical practice, provided that the aforementioned species differences are considered and overcome.

### 4.3 Advantages and Limitations of This Study

#### 4.3.1 Advantages of This Study

1) Based on animal experiments, the real effects and limitations of NSCs in repairing SCI were systematically evaluated and analyzed in subgroups, and existing problems and direction for improvement were pointed out in the current field. 2) The results of NSCs repairing SCI were analyzed at different time points, and the effect of stem cells in the whole treatment process was studied more comprehensively. 3) In the absence of evidence for direct comparison, the optimal transplantation timings of stem cells were obtained by a network meta-analysis. 4) Based on the internationally recognized SYRCLE bias risk assessment tool, the internal bias risk of animal experiments was strictly evaluated, and the problems in the design and implementation of animal experiments in this field were pointed out. At the same time, suggestions on how to improve the quality of animal experiments were given.

#### 4.3.2 Limitations of This Study

1) Although there is a certain basis for data selection based on the recovery of motor function and inflammatory response in SCI rats, whether the data selection method is reliable is still uncertain. 2) In the absence of unified staging and dose standards, we now consider the most reliable way to divide the transplantation doses and timings based on the previous literature and included studies. However, the rationality and scientific nature of this approach are still questionable. 3) In view of the fact that the regeneration of axons does not always mean the restoration of function (60), we only chose the BBB score, which is commonly reported and can directly reflect motor function as our outcome indicator, and did not include more outcome indicators. 4) In order to make our findings more specific, we performed subgroup analysis on the rat injury model, stem cell transplantation timing, and transplantation dose of stem cells. Although the results obtained from different subgroups were highly consistent, the small number of studies in each subgroup reduced the reliability of our results to a certain extent. 5) We cannot accurately identify the sources of heterogeneity. Therefore, we use the random-effects model for meta-analysis, making our conclusions more conservative. 6) Only Chinese and English databases were retrieved, which may lead to a certain language bias. 7) Gray literature and conference abstracts were not searched, potentially leading to the generation of publication bias.

## 5 Conclusion

Through the comprehensive analysis of the 39 studies included, we found that in the subgroups of animal models, measurement time point, and transplantation doses, the same results were obtained by the network meta-analysis, that is, NSC transplantation in the subacute phase can achieve the best therapeutic effect in rats with SCI. However, the results of the traditional meta-analysis were inconsistent, that is, in the moderate-dose group of moderate SCI model and the low-dose group of severe SCI model, transplantation in the subacute phase did not significantly improve motor function. Therefore, more studies, especially the evidence for direct comparison, will be needed in the future to further explore the optimal transplantation phase.

As the basis for the design and implementation of subsequent clinical trials, the quality of preclinical research directly determines whether the research results can be transformed into clinical practice. Through the comprehensive analysis of the bias risk, internal authenticity, and external authenticity of the included studies, we believe that there are still certain problems in the current animal experiments in terms of random grouping, allocation concealment, blinding method, and measurement and reporting of results. Especially for the BBB score that is dependent on subjective evaluation, these problems can seriously reduce the quality of animal experiments. Therefore, future studies need to further standardize the implementation and reporting of animal experiments, so as to improve the quality of evidence in preclinical studies and reduce the risk of preclinical research results transforming to clinical practice.

## Data Availability Statement

The original contributions presented in the study are included in the article/**Supplementary Material**. Further inquiries can be directed to the corresponding authors.

## Author Contributions

XW and PW undertook the design, guidance, and modification of the project and paper. ZS completed the implementation of the project and writing of the paper, and other authors completed the collection and collation of the data. All authors contributed to the article and approved the submitted version.

## Funding

This work was supported by the Gansu Natural Science Foundation (No. 21JR7RA362; 21JR7RA413) and The First Hospital of Lanzhou University Foundation (No. ldyyyn2021-121).

## Conflict of Interest

The authors declare that the research was conducted in the absence of any commercial or financial relationships that could be construed as a potential conflict of interest.

## Publisher’s Note

All claims expressed in this article are solely those of the authors and do not necessarily represent those of their affiliated organizations, or those of the publisher, the editors and the reviewers. Any product that may be evaluated in this article, or claim that may be made by its manufacturer, is not guaranteed or endorsed by the publisher.
