# Supplementary material for: What Is the Optimal Timing of Transplantation of Neural Stem Cells in Spinal Cord Injury? A Systematic Review and Network Meta-Analysis Based on Animal Studies
Source: Front Immunol. 2022 Mar 10;13:855309. doi: 10.3389/fimmu.2022.855309 (PMC8965614; doi:10.3389/fimmu.2022.855309)
Supplement: Supplementary file 1 [file Table_1.docx]

**What is the Optimal Timing of Transplantation of Neural Stem Cells in Spinal Cord Injury? A Systematic Review and Network Meta-analysis Based on Animal Studies**

**Table 1: Chinese and English search strategies**

| **PubMed**  #1: "Neural Stem Cells"[MeSH Major Topic] 9108  #2: "neural stem cell"[Title/Abstract] OR "nerve stem cell"[Title/Abstract] OR "neuronal stem cells"[Title/Abstract] 5444  #3: "stem cell"[Title/Abstract] AND ("nerve"[Title/Abstract] OR "nerval"[Title/Abstract] OR "neurologic"[Title/Abstract] OR "neural"[Title/Abstract]) 14313  #4: #1 OR #2 OR #3 20279  #5: "Spinal Cord Injuries"[MeSH Terms] 52034  #6: "spinal cord injury"[Title/Abstract] OR "spinal injury"[Title/Abstract] OR "spinal cord trauma"[Title/Abstract] OR "spinal cord transection"[Title/Abstract] OR "spinal cord laceration"[Title/Abstract] OR "post traumatic myelopathy"[Title/Abstract] OR "spinal cord contusion"[Title/Abstract] 42373  #7: #5 OR #6 65881  #8: #4 AND #7 1069  **Web of science**  ((TS= (neural stem cell OR nerve stem cell OR neuronal stem cells)) OR TS= (stem cell AND (nerve OR nerval OR neurologic OR neural))) AND TS= (Spinal cord injury OR Spinal injury OR Spinal Cord Trauma OR Spinal Cord Transection OR Spinal Cord Laceration OR Post-Traumatic Myelopathy OR Spinal Cord Contusion) 7521  **Embase**  #1: (neural stem cell or nerve stem cell or neuronal stem cells or (stem cell and (nerve or nerval or neurologic or neural))).mp. [mp=title, abstract, heading word, drug trade name, original title, device manufacturer, drug manufacturer, device trade name, keyword heading word, floating subheading word, candidate term word] 75347  #2: neural stem cell/ 28656  #3: stem cell/ 133372  #4: nerve/ 19603  #5: 3 and 4 279  #6: 1 or 2 or 5 75347  #7: (Spinal cord injury or Spinal injury or Spinal Cord Trauma or Spinal Cord Transection or Spinal Cord Laceration or Post-Traumatic Myelopathy or Spinal Cord Contusion).mp. [mp=title, abstract, heading word, drug trade name, original title, device manufacturer, drug manufacturer, device trade name, keyword heading word, floating subheading word, candidate term word] 64199  #8: spinal cord injury/ 50755  #9: spinal cord transsection/ 2226  #10: 7 or 8 or 9 64929  #11: 6 and 10 3737  **CNKI / Advanced Search**  Subject: spinal cord injury AND (neural stem cell OR neural stem cells) (1037)  **Wanfang database/ Advanced Search**  Subject: spinal cord injury AND (neural stem cell OR neural stem cells) (1388)  **VIP database/ Advanced Search**  Title or keyword: spinal cord injury AND (neural stem cell OR neural stem cells) (809)  **CBM / Advanced Search**  #1:" Spinal cord injury "[unweighted, extended] 60849  #2:" Spinal cord injury "[ common field: smart] 78804  #3: #2 OR #3 78804  #4:"Stem cell"[unweighted, extended] 356375  #5:" Stem cell "[ common field: smart] OR " stem cells"[ common field: smart] OR " progenitor cell "[ common field: smart] 677952  #6: #4 OR #5 677952  #7: #3 AND #6 1377 |
| --- |

**Table 2: Basic information of included studies**

| **No.** | **Author+year** | **Country** | **Type of study** | **Species** | **Gender** | **Weight** | **Age** | **Sample** | **Modeling method** | **Model type** | **Source** | **Transplantation route** | **Transplantation time** | **Transplantation dose** | **Control** |
| --- | --- | --- | --- | --- | --- | --- | --- | --- | --- | --- | --- | --- | --- | --- | --- |
| 1 | Stewart 2017[1] | USA | Control | SD rats | Female | 250-350g | 12-16weeks | 11/16 | NYU MASCIS Model II Impactor (10g) falls from 25mm height to dura at T8 level | Contusion | Sd rat fetus | Intralesional | 9days | 3×10^5^ | Hanks Buffered Saline Solution |
| 2 | Mohammadshirazi 2019[2] | Iran | RCT | Wistar rats | Male | 250-280g | Adult | 9/12 | NYU-impactor (10g) falls from 25mm height to dura at T8 level | Contusion | Human | Intralesional | 7days | 1×10^6^ | Blank |
| 3 | Pang 2019[3] | China | RCT | Long-Evans hooded rats | Male | / | 11weeks | 6/6 | NYU-impactor (10g) falls from 25mm height to dura at T9-T10 levels | Contusion | Human wisdom teeth | Intralesional | 7days | 1×10^6^ | Blank |
| 4 | Abdolahi 2021[4] | Iran | RCT | Wistar rats | Male | 220-250g | Adult | 8/8 | Place 35 g weight on the dura mater of T9-T10 for 15 minutes | Compression | Epileptic | Intralesional | 10days | 1×10^4^ | PBS |
| 5 | Amemori 2013[5] | Czech Republic | RCT | Wistar rats | Male | 270-300g | 10weeks | 20/16 | Balloon compression at T8-T9 levels with 15 μl saline for 5 minutes | Compression | Human fetal spinal cord | Intralesional | 7days | 5×10^5^ | Saline |
| 6 | Fan 2017[6] | China | RCT | SD rats | Female | / | / | 25/25 | The spinal cord was compressed using a calibrated aneurysm clip for 60s, which provided 20 g/cm2 pressure at T9-T11. | Compression | Sd rat fetus | Intralesional | 0days | 2×10^5^ | DMEM |
| 7 | Deng 2021[7] | China | RCT | SD rats | Female | 200-250g | 8weeks | 8/8 | Impact rod (10g) falling from 25mm height to dura at T7 level | Contusion | Hippocampus of neonatal SD rats | Intralesional | 0days | 1×10^6^ | Saline |
| 8 | Hong 2014[8] | Korea | RCT | SD rats | Female | 230-250g | 12weeks | 24/23 | NYU MASCIS Model II Impactor (10g) falls from 25mm height to dura at T9 level | Contusion | Sd rat fetus | Intralesional | 9days | 1×10^6^ | PBS |
| 9 | Hwang 2014[9] | Korea | RCT | SD rats | Male | 250-300g | / | 8/8 | A contusion injury at T9 using the Infinite Horizon Impactor (200 kdyn). | Contusion | SD rats | Intralesional | 7days | 5×10^5^ | PBS |
| 10 | Zhang 2015[10] | China | RCT | Wistar rats | Male | 200±10g | 8weeks | 20/20 | NYU-impactor (10g) falls from 12.5mm height to dura at T10 | Contusion | Brain tissue of Wistar rats | Intralesional | 3days | 1.5×10^5^ | Blank |
| 11 | Ormond 2014[11] | USA | RCT | SD rats | Female | 200-250g | Adult | 9/12 | NYU-impactor (10g) falls from 12.5mm height to dura at T10 | Contusion | SD rats | Intralesional | 7days | 1×10^6^ | DMSO |
| 12 | Shao 2021[12] | China | RCT | Wistar rats | / | / | 8-9weeks | 12/12 | NYU-impactor (5g) falls from 20mm height to dura at T10 | Contusion | Human | Intralesional | 7days | 1×10^5^ | Saline |
| 13 | Song 2015[13] | China | RCT | SD rats | Female | / | / | 20/20 | Allen method | Contusion | Sd rat fetus | Intralesional | 6h | 1×10^5^ | PBS |
| 14 | Li 2014[14] | China | RCT | SD rats | Male and Female | 240±10g | 10weeks | 18/18 | NYU-impactor (10g) falls from 25mm height to dura at T10 level | Contusion | Human aborted embryos | Intralesional | 0h | 2×10^5^ | Saline |
| 15 | Watanabe 2004[15] | Japan | RCT | SD rats | Female | 230-250g | Adult | 18/10 | NYU-impactor (10g) falls from 25mm height to dura at T10 level | Contusion | Sd rat fetus | Intralesional | 9days | 5×10^5^ | DMEM |
| 16 | Zarei-Kheirabadi 2020[16] | Iran | RCT | Wistar rats | Male | 250-280g | Adult | 10/10 | NYU-impactor (10g) falls to dura at T10-T11 levels from 25mm height | Contusion | Human aborted embryos | Intralesional | 7days | 1×10^6^ | PBS |
| 17 | Wu 2015[17] | China | RCT | SD rats | Female | 200-250g | / | 15/10 | Injury of T10 spinal cord with NYU MASCIS impactor ( 10 g × 50 mm ) | Contusion | Abortion embryo of cynomolgus monkey | Intralesional | 0h | 2.5×10^4^ | Blank |
| 18 | Cheng 2012[18] | USA | RCT | Long-Evans hooded rats | Female | 200-350g | 11weeks | 6/6 | NYU-impactor (10g) falls from 25mm height to dura at T8 level | Contusion | Human aborted embryos | Intralesional | 0h | 5×10^5^ | DMEM |
| 19 | Wu 2011[19] | China | RCT | SD rats | Female | 250-280g | / | 40/40 | Model of T8 spinal cord compression injury | Compression | Sd rat fetus | Intralesional | 7days | 3.5×10^5^ | Saline |
| 20 | Zhou 2001[20] | China | RCT | SD rats | Male and Female | 180-237g | Adult | 20/20 | T10 spinal cord injury in rats induced by Allen ' s strike rod position (2.5 g × 10 cm) | Contusion | SD rats | Intralesional | 7days | 1×10^6^ | Blank |
| 21 | Wang 2011[21] | China | RCT | Wistar rats | Male | 200-250g | Adult | 14/14 | NYU-impactor (10g) falls from 25mm height to dura at T10 level | Contusion | Human aborted embryos | Intralesional | 9days | 1×10^5^ | PBS |
| 22 | He 2010[22] | China | RCT | SD rats | / | 225±25g | / | 12/12 | A model of T10 spinal cord injury was established by free fall of a 10g hammer from 5cm height. | Contusion | Human aborted embryos | Intralesional | 8days | 5×10^4^ | DMEM |
| 23 | Zhong 2012[23] | China | RCT | SD rats | Male and Female | 230-250g | Adult |  | Establishment of T10 spinal cord injury model in rats by Allen method | Contusion | Spinal cord of SD rats | Intralesional | 0h | 5×10^5^ | Saline |
| 24 | Kong 2009[24] | China | RCT | SD rats | / | 250±20g | / | 10/10 | Establishment of T10 spinal cord injury model in rats by Allen method | Contusion | Sd rat fetus | Intralesional | 3days | 1×10^6^ | Saline |
| 25 | Zhang 2008[25] | China | RCT | Wistar rats | Male | 250-300g | Adult | 30/30 | NYU-impactor (10g) falls from 25mm height to dura at T9 level | Contusion | Sd rat fetus | Intralesional | 9days | 5×10^5^ | Blank |
| 26 | Zhao 2013[26] | China | RCT | SD rats | Female | 250-300g | 10weeks | 20/20 | Modified Allen method | Contusion | Sd rat fetus | Intralesional | 6h | 1×10^5^ | Blank |
| 27 | Wu 2016[27] | China | RCT | SD rats | / | 220-235g | 4weeks | 20/20 | Drop the weight vertically from 2.5 cm height and hit the spinal cord directly at 25 g · cm | Contusion | Sd rat fetus | Intralesional | 6h | 1×10^5^ | Saline |
| 28 | Zhao 2019[28] | China | RCT | SD rats | / | 180-220g | Adult | 6/6 | Transection at T10 | Transection | Hippocampus of neonatal SD rats | Intralesional | 7days | 1×10^5^ | PBS |
| 29 | Du 2014[29] | China | RCT | SD rats | Female | 220-250g | / | 6/6 | Transection at T10 | Transection | Hippocampus of neonatal SD rats | Intralesional | 0h | 1×10^6^ | Blank |
| 30 | Gu 2012[30] | China | RCT | SD rats | / | / | Adult | 25/25 | Transection at T8 | Transection | embryonic mouse | Intralesional | 0h | 3×10^5^ | Blank |
| 31 | He 2013[31] | China | RCT | SD rats | / | / | Adult | 25/25 | Transection atT10 | Transection | embryonic mouse | Intralesional | 0h | 3×10^5^ | Blank |
| 32 | Li 2011[32] | China | RCT | SD rats | Female | 200-250g | Adult | 5/5 | Transection atT9 | Transection | embryonic mouse | Intralesional | 0and7days | 6×10^5^ | Blank |
| 33 | Le 2014[33] | China | RCT | SD rats | Female | 180-220g | Adult | 27/6 | Transection atT10 | Transection | Sd rat fetus | Intralesional | 1h | 1×10^6^ | DMEM |
| 34 | Yuan 2019[34] | China | RCT | SD rats | Female | 220-250g | Adult | 30/25 | Transection atT10 | Transection | Sd rat fetus | Intralesional | 0h | 1.5×10^5^ | Saline |
| 35 | Zhao 2007[35] | China | RCT | SD rats | Female | / | Adult | 9/5 | Transection atT10 | Transection | Hippocampus of neonatal SD rats | Intralesional | 0h | 1×10^6^ | DMEM |
| 36 | Wu 2014[36] | China | RCT | SD rats | Female | 180-220g | Adult | 20/20 | Transection atT10 | Transection | Maternal umbilical cord blood | Intralesional | 7days | 1×10^7^ | PBS |
| 37 | Yin 2006[37] | China | RCT | Wistar rats | Male | 250-300g | Adult | 12/12 | Transection atT10 | Transection | Human aborted embryos | Intralesional | 9days | 2.5×10^5^ | DMEM |
| 38 | Li 2004[38] | China | RCT | Wistar rats | / | 250±50g | Adult | 10/10 | Transection atL4 | Transection | Wistar rats | Intralesional | 0h | 5×10^5^ | Blank |
| 39 | Liu 2011[39] | China | RCT | SD rats | Male | / | / | 10/10 | Transection atT10 | Transection | Hippocampus of neonatal SD rats | Intralesional | 7days | 1×10^4^ | DMEM |

**Table 3: Traditional meta-analysis result**

| **Model** | **Transplantation dose** | **Measure time (week)** | **Transplantation time** | **Number of studies** | **WMD** |
| --- | --- | --- | --- | --- | --- |
| Moderate injury | High | 1 | Acute_phase | 2 | 0.63[-0.06, 1.31] |
| Moderate injury | High | 1 | Subacute_phase | 5 | 1.20[0.15, 2.26] |
| Moderate injury | High | 1 | Chronic_phase | 1 | 0.38[-0.41, 1.17] |
| Moderate injury | High | 3 | Acute_phase | 2 | 2.53[-0.56, 5.62] |
| Moderate injury | High | 3 | Subacute_phase | 5 | 2.68[1.96, 3.41] |
| Moderate injury | High | 3 | Chronic_phase | 1 | 0.59[0.03, 1.15] |
| Moderate injury | High | 5 | Acute_phase | 1 | 2.08[1.45, 2.71] |
| Moderate injury | High | 5 | Subacute_phase | 5 | 2.66[1.54, 3.79] |
| Moderate injury | High | 5 | Chronic_phase | 1 | -0.24[-0.78, 0.30] |
| Moderate injury | High | 8 | Acute_phase | 1 | 3.03[2.38, 3.68] |
| Moderate injury | High | 8 | Chronic_phase | 1 | 0.65[0.12, 1.18] |
| Moderate injury | Moderate | 1 | Acute_phase | 4 | 1.86[-0.62, 4.34] |
| Moderate injury | Moderate | 1 | Subacute_phase | 3 | 4.10[-3.62, 11.82] |
| Moderate injury | Moderate | 1 | Chronic_phase | 3 | 1.44[0.32, 2.56] |
| Moderate injury | Moderate | 3 | Acute_phase | 5 | 4.25[1.90, 6.61] |
| Moderate injury | Moderate | 3 | Subacute_phase | 3 | 4.81[-2.27, 11.89] |
| Moderate injury | Moderate | 3 | Chronic_phase | 3 | 2.96[0.23, 5.69] |
| Moderate injury | Moderate | 5 | Acute_phase | 1 | 1.86[0.67, 3.05] |
| Moderate injury | Moderate | 5 | Subacute_phase | 2 | 2.73[1.95, 3.51] |
| Moderate injury | Moderate | 5 | Chronic_phase | 2 | 1.77[-1.20, 4.73] |
| Moderate injury | Moderate | 8 | Acute_phase | 1 | 6.29[5.11, 7.47] |
| Moderate injury | Moderate | 8 | Subacute_phase | 2 | 6.92[-0.69, 14.52] |
| Moderate injury | Moderate | 8 | Chronic_phase | 1 | 1.96[1.30, 2.62] |
| Moderate injury | Low | 1 | Acute_phase | 4 | 0.81[0.63, 0.98] |
| Moderate injury | Low | 1 | Subacute_phase | 1 | 0.94[0.51, 1.37] |
| Moderate injury | Low | 1 | Chronic_phase | 3 | 0.66[-0.83, 2.14] |
| Moderate injury | Low | 3 | Acute_phase | 4 | 2.04[1.93, 2.16] |
| Moderate injury | Low | 3 | Subacute_phase | 1 | 2.89[2.03, 3.75] |
| Moderate injury | Low | 3 | Chronic_phase | 3 | 1.53[-0.38, 3.43] |
| Moderate injury | Low | 5 | Acute_phase | 3 | 4.90[4.50, 5.29]] |
| Moderate injury | Low | 5 | Chronic_phase | 3 | 2.54[0.89, 4.19] |
| Moderate injury | Low | 8 | Acute_phase | 3 | 4.32[4.22, 4.42] |
| Severe injury | High | 1 | Acute_phase | 2 | 0.54[-0.02, 1.10] |
| Severe injury | High | 1 | Subacute_phase | 1 | 0.65[0.20, 1.10] |
| Severe injury | High | 3 | Acute_phase | 3 | 2.03[0.33, 3.72] |
| Severe injury | High | 3 | Subacute_phase | 1 | 2.03[1.56, 2.50] |
| Severe injury | High | 5 | Acute_phase | 2 | 1.26[1.03, 1.49] |
| Severe injury | High | 5 | Subacute_phase | 1 | 2.87[2.24, 3.50] |
| Severe injury | High | 8 | Acute_phase | 2 | 2.71[1.27, 4.15] |
| Severe injury | High | 8 | Subacute_phase | 1 | 6.17[5.25, 7.09] |
| Severe injury | Moderate | 1 | Acute_phase | 3 | 0.08[0.03, 0.13] |
| Severe injury | Moderate | 1 | Subacute_phase | 1 | 1.31[0.74, 1.88] |
| Severe injury | Moderate | 3 | Acute_phase | 4 | 1.29[1.00, 1.58] |
| Severe injury | Moderate | 3 | Chronic_phase | 1 | 0.75[0.33, 1.17] |
| Severe injury | Moderate | 5 | Acute_phase | 1 | 3.01[2.64, 3.38] |
| Severe injury | Moderate | 5 | Chronic_phase | 1 | 1.60[0.89, 2.31] |
| Severe injury | Moderate | 8 | Acute_phase | 2 | 6.19[2.85, 9.52] |
| Severe injury | Moderate | 8 | Chronic_phase | 1 | 3.30[2.41, 4.19] |
| Severe injury | Low | 1 | Subacute_phase | 2 | 0.25[-0.34, 0.83] |
| Severe injury | Low | 3 | Subacute_phase | 1 | 2.33[1.47, 3.19] |
| Severe injury | Low | 5 | Subacute_phase | 1 | 3.83[2.97, 4.69] |

**Reference**

1. Stewart AN, Kendziorski G, Deak ZM, Brown DJ, Fini MN, Copely KL, Rossignol J, Dunbar GL. Co-transplantation of mesenchymal and neural stem cells and overexpressing stromal-derived factor-1 for treating spinal cord injury. Brain research. 2017 Oct 1;1672:91-105. eng. Epub 2017/07/25. doi:10.1016/j.brainres.2017.07.005. Cited in: Pubmed; PMID 28734802.

2. Mohammadshirazi A, Sadrosadat H, Jaberi R, Zareikheirabadi M, Mirsadeghi S, Naghdabadi Z, Ghaneezabadi M, Fardmanesh M, Baharvand H, Kiani S. Combinational therapy of lithium and human neural stem cells in rat spinal cord contusion model. Journal of cellular physiology. 2019 Nov;234(11):20742-20754. eng. Epub 2019/04/21. doi:10.1002/jcp.28680. Cited in: Pubmed; PMID 31004353.

3. Pang CY, Yang KL, Fu CH, Sun LY, Chen SY, Liao CH. G-CSF enhances the therapeutic potency of stem cells transplantation in spinal cord-injured rats. Regenerative medicine. 2019 Jun;14(6):571-583. eng. Epub 2019/05/23. doi:10.2217/rme-2018-0104. Cited in: Pubmed; PMID 31115255.

4. Abdolahi S, Aligholi H, Khodakaram-Tafti A, Khaleghi Ghadiri M, Stummer W, Gorji A. Improvement of Rat Spinal Cord Injury Following Lentiviral Vector-Transduced Neural Stem/Progenitor Cells Derived from Human Epileptic Brain Tissue Transplantation with a Self-assembling Peptide Scaffold. Molecular neurobiology. 2021 Jun;58(6):2481-2493. eng. Epub 2021/01/15. doi:10.1007/s12035-020-02279-5. Cited in: Pubmed; PMID 33443682.

5. Amemori T, Romanyuk N, Jendelova P, Herynek V, Turnovcova K, Prochazka P, Kapcalova M, Cocks G, Price J, Sykova E. Human conditionally immortalized neural stem cells improve locomotor function after spinal cord injury in the rat. Stem cell research & therapy. 2013 Jun 7;4(3):68. eng. Epub 2013/06/14. doi:10.1186/scrt219. Cited in: Pubmed; PMID 23759119.

6. Fan WL, Liu P, Wang G, Pu JG, Xue X, Zhao JH. Transplantation of hypoxic preconditioned neural stem cells benefits functional recovery via enhancing neurotrophic secretion after spinal cord injury in rats. Journal of cellular biochemistry. 2018 Jun;119(6):4339-4351. eng. Epub 2017/09/09. doi:10.1002/jcb.26397. Cited in: Pubmed; PMID 28884834.

7. Deng M, Xie P, Chen Z, Zhou Y, Liu J, Ming J, Yang J. Mash-1 modified neural stem cells transplantation promotes neural stem cells differentiation into neurons to further improve locomotor functional recovery in spinal cord injury rats. Gene. 2021 May 20;781:145528. eng. Epub 2021/02/26. doi:10.1016/j.gene.2021.145528. Cited in: Pubmed; PMID 33631250.

8. Hong JY, Lee SH, Lee SC, Kim JW, Kim KP, Kim SM, Tapia N, Lim KT, Kim J, Ahn HS, Ko K, Shin CY, Lee HT, Schöler HR, Hyun JK, Han DW. Therapeutic potential of induced neural stem cells for spinal cord injury. The Journal of biological chemistry. 2014 Nov 21;289(47):32512-25. eng. Epub 2014/10/09. doi:10.1074/jbc.M114.588871. Cited in: Pubmed; PMID 25294882.

9. Hwang DH, Shin HY, Kwon MJ, Choi JY, Ryu BY, Kim BG. Survival of neural stem cell grafts in the lesioned spinal cord is enhanced by a combination of treadmill locomotor training via insulin-like growth factor-1 signaling. The Journal of neuroscience : the official journal of the Society for Neuroscience. 2014 Sep 17;34(38):12788-800. eng. Epub 2014/09/19. doi:10.1523/jneurosci.5359-13.2014. Cited in: Pubmed; PMID 25232115.

10. Zhang C, Shen L. Folic acid in combination with adult neural stem cells for the treatment of spinal cord injury in rats. International journal of clinical and experimental medicine. 2015;8(7):10471-80. eng. Epub 2015/09/18. Cited in: Pubmed; PMID 26379837.

11. Ormond DR, Shannon C, Oppenheim J, Zeman R, Das K, Murali R, Jhanwar-Uniyal M. Stem cell therapy and curcumin synergistically enhance recovery from spinal cord injury. PloS one. 2014;9(2):e88916. eng. Epub 2014/02/22. doi:10.1371/journal.pone.0088916. Cited in: Pubmed; PMID 24558450.

12. Shao R, Zhang L, Yang H, Wang Y, Zhang Z, Yue J, Chen Y, Pan H, Zhou H, Quan R. Autophagy activation promotes the effect of iPSCs-derived NSCs on bladder function restoration after spinal cord injury. Tissue & cell. 2021 Oct;72:101596. eng. Epub 2021/08/02. doi:10.1016/j.tice.2021.101596. Cited in: Pubmed; PMID 34333229.

13. Song YY, Peng CG, Ye XB. Combination of edaravone and neural stem cell transplantation repairs injured spinal cord in rats. Genetics and molecular research : GMR. 2015 Dec 29;14(4):19136-43. eng. Epub 2016/01/20. doi:10.4238/2015.December.29.23. Cited in: Pubmed; PMID 26782566.

14. Li Z, Zhao W, Liu W, Zhou Y, Jia J, Yang L. Transplantation of placenta-derived mesenchymal stem cell-induced neural stem cells to treat spinal cord injury. Neural regeneration research. 2014 Dec 15;9(24):2197-204. eng. Epub 2015/02/07. doi:10.4103/1673-5374.147953. Cited in: Pubmed; PMID 25657742.

15. Watanabe K, Nakamura M, Iwanami A, Fujita Y, Kanemura Y, Toyama Y, Okano H. Comparison between fetal spinal-cord- and forebrain-derived neural stem/progenitor cells as a source of transplantation for spinal cord injury. Developmental neuroscience. 2004 Mar-Aug;26(2-4):275-87. eng. Epub 2005/02/16. doi:10.1159/000082144. Cited in: Pubmed; PMID 15711067.

16. Zarei-Kheirabadi M, Sadrosadat H, Mohammadshirazi A, Jaberi R, Sorouri F, Khayyatan F, Kiani S. Human embryonic stem cell-derived neural stem cells encapsulated in hyaluronic acid promotes regeneration in a contusion spinal cord injured rat. International journal of biological macromolecules. 2020 Apr 1;148:1118-1129. eng. Epub 2020/01/27. doi:10.1016/j.ijbiomac.2020.01.219. Cited in: Pubmed; PMID 31982534.

17. Wu MF, Zhang SQ, Gu R, Liu JB, Li Y, Zhu QS. Transplantation of erythropoietin gene-modified neural stem cells improves the repair of injured spinal cord. Neural regeneration research. 2015 Sep;10(9):1483-90. eng. Epub 2015/11/26. doi:10.4103/1673-5374.165521. Cited in: Pubmed; PMID 26604911.

18. Cheng I, Mayle RE, Cox CA, Park DY, Smith RL, Corcoran-Schwartz I, Ponnusamy KE, Oshtory R, Smuck MW, Mitra R, Kharazi AI, Carragee EJ. Functional assessment of the acute local and distal transplantation of human neural stem cells after spinal cord injury. The spine journal : official journal of the North American Spine Society. 2012 Nov;12(11):1040-4. eng. Epub 2012/10/16. doi:10.1016/j.spinee.2012.09.005. Cited in: Pubmed; PMID 23063425.

19. Wu QL, Liang JW, Yan XL, Sun YL, Huang HL, Liu K. Neural stem cells transplantation combined with monosialotetrahexosyl ganglioside for treatment of spinal cord injury in rats. Chinese Journal of Trauma. 2011;(09):834-838. [Article in Chinese]

20. Zhou ZL, Jin AM, Chen YH, Ming SX. Effects of rehabilitation training combined with bone marrow mesenchymal stem cell-derived neural stem cells transplantation on the expression of Nogo-A and NgR proteins after spinal cord injury. Chinese Journal of Physical Medicine and Rehabilitation. 2011;(08):566-570. [Article in Chinese]

21. Wang P, Wu YF, Meng QQ, Huang L, Wang WH, Gao LB, Tang Y, Shen HL. The influence and mechanism of delayed transplantation of human fetal neural stem cells on locomotor recovery after experimental spinal cord injury in rats. Chinese Journal of Spine and Spinal Cord. 2011;21(11):934-940. [Article in Chinese]

22. He XY, Shen HY, Xiang P, Wu YF, Tang Y, Shi YP, Huang L, Yang R, Ye JC. Effects of human neural stem cell transplantation on the recovery of hindlimb motor function in rats with spinal cord injury. Chinese Journal of Sports Medicine. 2010;29(02):208-210. [Article in Chinese]

23. Zhong L, Zhang H, Gao WL, Yin ZS. Comparative study on single cell suspenion of neural stem cells and neurospheres transplantation for spinal cord injury in rats. Chinese Journal of Reparative and Reconstructive Surgery. 2012;26(07):855-860. [Article in Chinese]

24. Kong LS, Nie DL, Zhang JC, Zhang H, Xu H. Experimental study of neural stem cell transplantation to promote the survival of anterior horn motor neurons and the recovery of hindlimb motor function after spinal cord injury in rats. The Journal of Practical Medicine. 2009;25(20):3402-3404. [Article in Chinese]

25. Zhang LF, Mao ZB, Jiang ZL. In vivo tracing study of neural stem cell transplantation in the treatment of spinal cord injury in rats. Journal of Southeast University(Medical Science Edition). 2008;(03):199-202+148. [Article in Chinese]

26. Zhao ZJ, Sun ZM, Zhang CY, Han JG, Shi RC, Wang Zw. Edaravone combined with neural stem cell transplantation for treatment of spinal cord injury in rats. Chinese Journal of Tissue Engineering Research. 2013;17(10):1862-1867. [Article in Chinese]

27. Wu YJ, Hou YN, Zhang ZT, Liu ZP, Nie ZH, Fan GL. Early exercise training combined with neural stem cell transplantation improves hindlimb motor function after spinal cord injury in rats. Chinese Journal of Tissue Engineering Research. 2016;20(06):876-882. [Article in Chinese]

28. Zhao XM, He XY, Liu J, Xu Y, Xu FF, Tan YX, Zhang ZB, Wang TH. Neural Stem Cell Transplantation Improves Locomotor Function in Spinal Cord Transection Rats Associated with Nerve Regeneration and IGF-1 R Expression. Cell transplantation. 2019 Sep-Oct;28(9-10):1197-1211. eng. Epub 2019/07/05. doi:10.1177/0963689719860128. Cited in: Pubmed; PMID 31271053.

29. Du BL, Zeng X, Ma YH, Lai BQ, Wang JM, Ling EA, Wu JL, Zeng YS. Graft of the gelatin sponge scaffold containing genetically-modified neural stem cells promotes cell differentiation, axon regeneration, and functional recovery in rat with spinal cord transection. Journal of biomedical materials research Part A. 2015 Apr;103(4):1533-45. eng. Epub 2014/07/22. doi:10.1002/jbm.a.35290. Cited in: Pubmed; PMID 25046856.

30. Gu YL, Yin LW, Zhang Z, Liu J, Liu SJ, Zhang LF, Wang TH. Neurotrophin expression in neural stem cells grafted acutely to transected spinal cord of adult rats linked to functional improvement. Cellular and molecular neurobiology. 2012 Oct;32(7):1089-97. eng. Epub 2012/05/11. doi:10.1007/s10571-012-9832-4. Cited in: Pubmed; PMID 22573254.

31. He BL, Ba YC, Wang XY, Liu SJ, Liu GD, Ou S, Gu YL, Pan XH, Wang TH. BDNF expression with functional improvement in transected spinal cord treated with neural stem cells in adult rats. Neuropeptides. 2013 Feb;47(1):1-7. eng. Epub 2012/09/11. doi:10.1016/j.npep.2012.06.001. Cited in: Pubmed; PMID 22959240.

32. Li Y, Zhang WM, Wang TH. Optimal location and time for neural stem cell transplantation into transected rat spinal cord. Cellular and molecular neurobiology. 2011 Apr;31(3):407-14. eng. Epub 2010/12/17. doi:10.1007/s10571-010-9633-6. Cited in: Pubmed; PMID 21161579.

33. Wang L, Wei FX, Cen JS, Ping SN, Li ZQ, Chen NN, Cui SB, Wan Y, Liu SY. Early administration of tumor necrosis factor-alpha antagonist promotes survival of transplanted neural stem cells and axon myelination after spinal cord injury in rats. Brain research. 2014 Aug 5;1575:87-100. eng. Epub 2014/06/03. doi:10.1016/j.brainres.2014.05.038. Cited in: Pubmed; PMID 24887643.

34. Yuan T, Liu Q, Kang J, Gao H, Gui S. High-Dose Neural Stem/Progenitor Cell Transplantation Increases Engraftment and Neuronal Distribution and Promotes Functional Recovery in Rats after Acutely Severe Spinal Cord Injury. Stem cells international. 2019;2019:9807978. eng. Epub 2019/10/01. doi:10.1155/2019/9807978. Cited in: Pubmed; PMID 31565061.

35. Zhang X, Zeng Y, Zhang W, Wang J, Wu J, Li J. Co-transplantation of neural stem cells and NT-3-overexpressing Schwann cells in transected spinal cord. Journal of neurotrauma. 2007 Dec;24(12):1863-77. eng. Epub 2007/12/28. doi:10.1089/neu.2007.0334. Cited in: Pubmed; PMID 18159998.

36. Wu YK, Wang SW, Sun QJ, Huo TJ, Ma JH, Guo HN, Qin JZ, Dai YW, Xu RX. Umbilical cord blood neural stem cells transplantation for treatment of spinal cord injury in rats and differential expression gene. Chinese Journal of Neuromedicine. 2014;13(08):772-777. [Article in Chinese]

37. Yin GD, Tang X, Lin YQ, Xu YQ, Zhou TH. Experimental study on transplantation of human embryonic neural stem cells to adult rats′ transected spinal cord. Chinese Journal of Spine and Spinal Cord. 2006;(08):611-614+643. [Article in Chinese]

38. Li CR, Li W, Cai WQ, Chen DY, Su BY. Effects of neural stem cell transplantation on the functional revovery of rats with spinal cord injury. Chinese Journal of Tissue Engineering Research. 2004;(29):6364-6366+6548. [Article in Chinese]

39. Liu N, HuDL, Xu T, Yu SH, Sheng WB. Neural stem cells transplantation for the treatment of spinal cord injury. Chinese Journal of Tissue Engineering Research. 2011;15(10):1809-1813. [Article in Chinese]
